# Enhanced Whitefly Resistance in Transgenic Tobacco Plants Expressing Double Stranded RNA of *v-ATPase A* Gene

**DOI:** 10.1371/journal.pone.0087235

**Published:** 2014-03-03

**Authors:** Nidhi Thakur, Santosh Kumar Upadhyay, Praveen C. Verma, Krishnappa Chandrashekar, Rakesh Tuli, Pradhyumna K. Singh

**Affiliations:** 1 Plant Molecular Biology Lab, National Botanical Research Institute, Lucknow, Uttar Pradesh, India; 2 Academy of Scientific and Innovative Research, Anusandhan Bhawan, NewDelhi, India; 3 Department of Biotechnology, National Agri-Food Biotechnology Institute, Mohali, Punjab, India; 4 Indian Agricultural Research Institute-Regional Station, Agricultural College Estate, Pune, Maharashtra, India; Natural Resources Canada, Canada

## Abstract

**Background:**

Expression of double strand RNA (dsRNA) designed against important insect genes in transgenic plants have been shown to give protection against pests through RNA interference (RNAi), thus opening the way for a new generation of insect-resistant crops. We have earlier compared the efficacy of dsRNAs/siRNAs, against a number of target genes, for interference in growth of whitefly (*Bemisia tabaci*) upon oral feeding. The *v*-ATPase subunit A (v-ATPaseA) coding gene was identified as a crucial target. We now report the effectiveness of transgenic tobacco plants expressing siRNA to silence *v-ATPaseA* gene expression for the control of whitefly infestation.

**Methodology/Principal Findings:**

Transgenic tobacco lines were developed for the expression of long dsRNA precursor to make siRNA and knock down the *v-ATPaseA* mRNA in whitefly. Molecular analysis and insecticidal properties of the transgenic plants established the formation of siRNA targeting the whitefly *v-ATPaseA*, in the leaves. The transcript level of *v-ATPaseA* in whiteflies was reduced up to 62% after feeding on the transgenic plants. Heavy infestation of whiteflies on the control plants caused significant loss of sugar content which led to the drooping of leaves. The transgenic plants did not show drooping effect.

**Conclusions/Significance:**

Host plant derived pest resistance was achieved against whiteflies by genetic transformation of tobacco which generated siRNA against the whitefly *v-ATPaseA* gene. Transgenic tobacco lines expressing dsRNA of *v-ATPaseA*, delivered sufficient siRNA to whiteflies feeding on them, mounting a significant silencing response, leading to their mortality. The transcript level of the target gene was reduced in whiteflies feeding on transgenic plants. The strategy can be taken up for genetic engineering of plants to control whiteflies in field crops.

## Introduction

Agricultural crops are damaged worldwide by more than 10,000 species of insects [1].To circumvent the yield losses due to insect attack, diverse approaches are evolving for genetic engineering of crops to impart host plant derived pest resistance [2], [3]. The δ-endotoxin proteins of *Bacillus thuringiensis* (Bt), have been deployed commercially to effectively control chewing insect pests in cotton and maize [4], [5], [6]. Large scale adoption of chewing pest resistant GM crops and significant reduction in the application of chemical pesticides has altered the species distribution of pests in agricultural fields [7], [8]. As a consequence, sap-sucking pests are now emerging as more serious pests in cotton fields in comparison to bollworms [1], [9]. This group of insect pests (jassid, whitefly, aphid, thrips, mealy bug, mites, mired bug etc.) not only damage plants by sucking photosynthetic assimilates, but also acts as vectors of several plant viruses [10]. Further, they excrete honey dew which promotes fungal infection, causing loss to the quality of farm produce [11], [12]. Due to host specificity of the δ-endotoxins, the Bt-crops are not effective against sap-sucking pests [13], [14].

The sweetpotato whitefly, *Bemisia tabaci* (*Gennadius*) (*Hemiptera: Aleyrodidae*), is a globally prevalent invasive sucking pest [15]. The species complex in genus *Bemisia* attacks more than 600 plant species [16]. Fast reproduction rate and quick ability to develop resistance to several chemical insecticides increase their invasive potential [17]. Hence, there is a need to search for alternative ways to control sucking pests. Mannose binding lectin of *Pinellia ternata* has been reported to provide protection against whitefly in tobacco, following its expression at a high level in the plastids [18]. However, such high levels of expression are not achievable by nuclear transformation, the chloroplast transformation technology is available for limited number of crops, and such high levels of expression are likely to adversely affect agronomic performance of field crops.

RNA interference (RNAi) operates through gene specific post transcriptional silencing [19], [20]. It is triggered by the presence of double stranded RNA (dsRNA) precursor molecules which are cleaved into 21–23 nucleotides long small interfering RNAs (siRNA) by the RNAi pathway, leading to the formation of single stranded guide RNAs. The guide strand is incorporated into RISC complex to finally base pair with the complementary sequence in target mRNA and induce its cleavage. This process spreads systemically in several eukaryotes, including insects, and has been reported in members belonging to orders *Lepidoptera, Coleoptera, Diptera, Hymenoptera* and *Hemiptera* [21]. Interference in the expression of the critical genes can lead to a variety of phenotypic effects ranging from growth reduction to mortality. The mechanism has strategically been utilised to develop transgenic plants resistant to viruses [22], parasitic nematodes [23] and some insects belonging to order *Coleoptera* [24], *Lepidoptera* [25] and *Hemiptera* [26], [27].

Injecting dsRNA in the body cavity of whitefly has been reported to induce RNAi which knocked down the expression of genes to 70% in midgut and salivary glands [28]. We have earlier reported RNAi mediated mortality of whitefly through oral delivery of dsRNA, targeting five important genes namely – *actin* ortholog, *ADP/ATP translocase*, *α-tubulin*, *ribosomal protein L9* (*rpl9*) and *v-ATPaseA*, which caused different degrees of mortality. The dsRNA targeting *v-ATPaseA* was most effective, with the LC_50_ of 3.08 µg/ml [29]. The efficacy of the delivery of dsRNA into whitefly fed on strategically designed transgenic plants has not yet been reported.

Vacuolar-type *ATPases* represent an evolutionarily conserved family of enzymes with diverse functions in eukaryotes. These are ubiquitously present in the membranes of intracellular compartments, like vacuoles, lysosomes, coated vesicles, secretary granules, and the trans-Golgi network. The enzyme pumps H^+^ (coupled with ATP hydrolysis) into the lumen of the organelles, leading to their acidification [30]. They are also found in the plasma membrane of many animal cell types and are involved in the pH homeostasis and membrane energization. The *v-ATPase* enzyme has also been reported in numerous ion-transporting insect epithelia [31]. It is a multi-subunit enzyme having two domains, V_1_ and V_0_. The subunit A of the V_1_ domain is the catalytic site, responsible for ATP hydrolysis. Suppression of subunit A of *v-ATPase* is expected to be lethal and we have demonstrated the same in whitefly in our previous study [29].

This study examines if transgenic tobacco plants, designed to express dsRNA precursor against the whitefly *v-ATPaseA*, can form sufficient siRNA and cause mortality to offer protection against the sucking pest. Validation of the mechanism and effectiveness of the protection can open new avenues for developing insect resistant transgenic crop plants.

## Materials and Methods

### Plant and Insect culture

Tobacco plants (*Nicotiana tabacum* L. cv. Petit Havana) were sown in soil mix consisting of peat, perlite and vermiculite (1∶1∶1) and grown in greenhouse at 26±2°C, 60–80% relative humidity (RH) and16 L: 8 D h photoperiod. The leaves were used for insect feeding assays and molecular analysis.

Whiteflies were initially collected from cotton field at NBRI, Lucknow, India and reared for several generations on potted cotton and tobacco plants in the insect growth chamber (26±2°C and RH 60–80%). The laboratory cultures reared on healthy potted seedlings were used for all the experiments.

### Construction of *v-ATPaseA* dsRNA expression cassette

**Figure 1:**
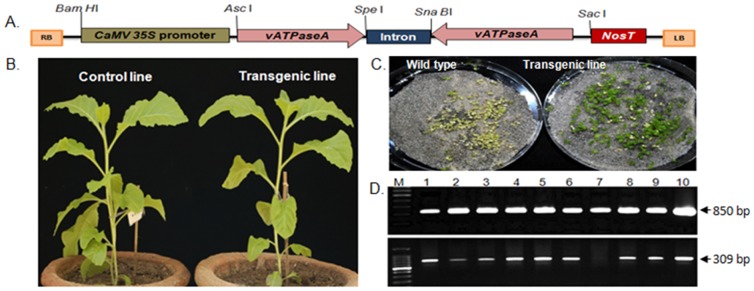
Development of transgenic tobacco expressing whitefly *v-ATPaseA* specific dsRNA. (**A**) Physical map of dsRNA expression cassette in pBI101. (**B**) transgenic and control tobacco plants showing comparable morphology. (**C**) selection of T_1_ seeds on kanamycin (300 mg/l) medium, showing non-transgenic seedlings turning white. (**D**) PCR analysis of transgenic lines in T_1_ generation by amplification of *nptII* gene (upper panel) and amplification of *v-ATPaseA*+*RTM1* intron (lower panel), M; 100 bp DNA ladder; lanes 2–9, different transgenic tobacco lines, lane 10; positive control.

The dsRNA expression cassette was constructed in pBlueScript SK+ (pSK+). The CaMVE35S promoter and 189 bp *v-ATPaseA* fragments were amplified, using primers that created the desired restriction sites at the ends. The amplified products were cloned at *Bam HI and Spe I* sites in pSK+. A 120 bp intron from *RTM1* gene of *Arabidopsis thaliana* was used as spacer. The intron and *v-ATPaseA* were amplified, using primers to create *Spe I* and *Sna BI* restriction sites. These were cloned at *Spe I* and *Sac I* sites of pSK+. In the resultant plasmid, *v-ATPaseA* fragment was ligated downstream of the intron in inverted orientation.The final dsRNA expression cassette [*Bam H1*-Promoter-vATPaseA-intron-vATPAseA (inverted)-*Sac I*] was cloned in the binary vector pBI101 at *Bam HI* and *Sac I* restriction sites and named as *pNT4*. The construct had CaMVE35S promoter, 189 bp *v-ATPaseA* fragment, an intron of 120 bp, 189 bp *v-ATPaseA* fragments in reverse orientation and *nos* terminator (Figure 1A). The 200 bp fragment of *asal* gene of *Allium sativum* (garlic) was cloned downstream of CaMVE35S promoter to serve as control in all the experiments. The primers used in making the constructs are listed in Table 1.

**Table 1:** Primers used in the study.

GENE	FORWARD PRIMER	REVERSE PRIMER
**GENE CLONING**		
*CaMVE 35S*	GATCTTAATTAACTGCAGGTCCGATTGAGA	ATGCGGCGCGCCGGATCTTGTAGAGAGAGACTG
*v-ATP*aseA sense strand	GATCGGCGCGCCTTTCCGGACGTTTGGCAGAGA	ATGCACTAGTGAGAAGTCACCACCAGGAGG
*v-ATP*aseA Antisense strand	GATCGAGCTCTTTCCGGACGTTTGGCAGAGA	ATGCTACGTAGAGAAGTCACCACCAGGAGG
*RTM* intron	GATCACTAGTGTAAGTCTGATTTTTGACTCTTC	ATGCTACGTACTGCTGGGTCCAAATCACATA
*nptII*	GAACAAGATGATTGCACGCA	GAAGAACTCGTCAAGAAGGC
*Asal* sense strand	GATCGGCGCGCCCAGGATGACTGCAAC	ATGCACTAGTTCCTCCTGAAGCAGCAGGATAT
*Asal* Antisense strand	GATCGAGCTCCAGGATGACTGCAAC	ATGCTACGTATCCTCCTGAAGCAGCAGGATAT
**RT-PCR**		
*v-ATP*aseA	ACCACCAGGAGGCCATACC	CCGGACGTTTGGCAGAGAT
*Actin*	GACCAGCCAAGTCCAAACGA	CCTTTGTGGTAGAGGTCTCAGTT

### Development of transgenic tobacco with *v-ATPaseA* dsRNA gene construct

*Agrobacterium tumefaciens* strain LBA4404 was transformed with *pNT4* through electroporation and used for the transformation of tobacco. Leaves were used as explants and transgenic plants were developed by direct organogenesis following standard procedure [32]. The transformants were selected on 300 mg/l kanamycin. Putative transgenic plants were grown in Magenta Boxes (Tarsons, India) at 26°C in the culture room and then transferred to the soil mix {peat, perlite and vermiculite (1∶1∶1)} and grown in glasshouse at 26±2°C for seed setting and generation advancement. T_1_ seeds were harvested and germinated on medium containing 300 mg/l kanamycin for segregation analysis and selection of transgenic plants. Another plant expression construct was developed in which the 189 bp *v-ATPaseA* fragment was replaced with a 200 bp fragment of *asal* gene that codes for the lectin of *Allium sativum* (accession number AY376827). Transgenic plants developed with this construct served as the source of dsRNA, taken as controls in all the experiments.

The insertion of T-DNA in transgenic lines was established by PCR, using the primers (Table 1) designed to amplify 850 bp *nptII* and 309 bp *v-ATPaseA*-intron fragment.

### Analysis of *v-ATPaseA* specific dsRNA in transgenic tobacco plants

Total RNA was isolated from young leaves of PCR positive twelve T_1_ transgenic and the control (*asal*) lines using TRI-reagent (Sigma, USA) according to manufacturer's protocol and used for dot-blot analysis. RNA samples (15 µg) were diluted in RNA denaturing buffer containing 66% formamide, 21% formaldehyde and 13% MOPS buffer and incubated at 65°C for 15 min for denaturation. Nylon membrane (Hybond-N^+^, GE-Amersham, UK) pre-wet in deionised water was equilibrated in 6× SSC buffer. Denatured RNA samples were spotted onto the membrane and cross linked by exposing to UV rays (300 nm). The dsRNA of *v-ATPaseA* (189 bp) was synthesised using Megascript RNAi kit (Ambion, USA) and labelled with AlkPhos direct labelling and detection system (GE-Amersham, UK) following manufacturer's protocol and used as probe. The snRNA probe U6 was used as internal control to ensure equal loading of the RNA samples. The hybridisation was carried out at 65°C for 6 h. After stringent washing, the blot was developed with substrate NBT-BCIP (Promega, USA).

### Northern blot analysis of transgenic plants

The presence of *v-ATPaseA* specific siRNA in transgenic tobacco plants was analysed by northern blot analysis. Small RNA was extracted from leaves of the T_1_ plants (control and transgenic lines 4, 7, 16 and 24) by mirVANA RNA isolation kit (Ambion, UK). Thirty µg per lane of RNA was resolved on 15% TBE-urea gel and blotted onto Hybond-N^+^ membrane (GE-Amersham, UK). The membrane was UV Cross-linked. Specific DNA probes were obtained by PCR using primers (as described for vector construction) and labelled with [α-32P] dCTP using Klenow fragment (Ambion, UK) and random primers. The blot was prehybridised at 60°C for 2 h and hybridisation was performed at 37°C for 16 h. The blot was washed with 2× SSC/0.2% SDS for 3×10 min at room temperature followed by 2× SSC/0.1% SDS for 10 min at 42°C. MicroRNA marker (NEB, UK) having 17, 21 and 25 residues of single-stranded RNA oligonucleotides was used as size standards to determine size of siRNA between 21–23 nucleotides. Loading of comparable amounts of RNA was assessed by ethidium bromide staining. The signals were detected after 72 h exposure to phosphor storage plates (BioRad, USA), scanned with a Typhoon TRIO+ scanner (GE Healthcare, UK) and analyzed using Image Quant™ (GE Healthcare).

### Whitefly toxicity assays

#### Leaf disc assay

Bioassay was carried out in 100 ml perforated specimen tubes in which 20–25 freshly hatched adult whiteflies were collected as described earlier [29].Twelve T_1_ transgenic lines were analysed. Two plants per transgenic line and three leaves per plant were tested in the bioassay. The caps of the vials were filled with 1% agar (up to 2/3). Fresh leaves from transgenic and control plants were placed on the gel and caps were placed on the whitefly carrying specimen tubes (Figure S2).The bioassay continued for 6 days. Whitefly cadavers were counted to calculate percent mortality. The average mortality in each transgenic line (2 plants×3 leaves per plant) was used for comparing means.

#### In-vivo bioassay of transgenic plants

Based on the RNA blot analysis and leaf disc bioassay, four transgenic lines (4, 7, 16, 24) were evaluated in comparison to the control in three different sets. Each experimental set consisted of two transgenic plants for each of the four lines and two control (*asal*) tobacco plants. Plants were randomly placed in a net chamber. Freshly hatched adult whiteflies were released on each plant individually. Each plant was covered with a poly bag at the time of releasing insects, which was removed once the insects settled on the plants. Initial count of the insects was taken. The position of the plants was changed every three days to negate possible position effect. Six plants per line and the same number of control plants were used for the study. The total number of whiteflies surviving on each plant was counted and the average number of insects on each line (two plants in each chamber X three different chambers) was used for calculation. The percent decrease in population over control was calculated by the formula of Fleming and Retnakaran [33].
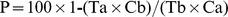
Where, P = % reduction in the number of whiteflies on transgenic plants over control, T_a_ = number of whiteflies surviving on transgenic plants after 15 days, T_b_ = number of whiteflies on transgenic plants just after infestation, T_a_ = number of whiteflies on control plants after 15 days, C_b_ = number of whiteflies on control plants just after infestation.

#### Small RNA feeding bioassay

Total RNA was isolated from the leaves of four selected T_1_ transgenic plants using TRI reagent. High molecular weight RNA was removed by precipitation with 5% PEG-8000 containing 0.5M NaCl. Low molecular weight RNA was recovered by precipitating with 2.5 volumes of absolute ethanol. The preparation had endogenous small RNAs along with the diced *v-ATPaseA* specific siRNA. Small RNA (15 µg) was mixed in liquid artificial diet (yeast extract 5%, and sucrose 30%) and fed to whiteflies by sandwiching between two layers of stretched parafilm [29]. The experiment was conducted on three plants of each of the four lines in three technical replicates. The bioassay was carried out for six days, and the diet was changed after three days.

### Estimation of sugar content in the leaves

Three-month-old plants of the four selected lines were challenged with whiteflies. Sugar content of the leaves was estimated by phenol sulphuric acid method [34]. Leaf sample (1.5 gm) was homogenised in 2.5 ml of 80% ethanol. The supernatant (2.5 ml) and phenol were mixed in equal volumes. Equal volume (5 ml) of concentrated sulphuric acid was added to the mixture and mixed. The samples were cooled to 20°C and absorbance was measured at 490 nm in Lambda 35 UV/Vis spectrometer (Perkin Elmer, USA). Dextrose L (1 mg/ml) was used for making standard plot. The sugar content in the samples was calculated in mg/g of the fresh weight of leaf tissue.

### Quantitative real-time PCR analysis

Freshly hatched adults were released on transgenic lines 7, 16 and the control plants (Three biological replicates per line and three control plants).Whiteflies collected from each line were pooled after 2, 4, 6 and 8 days. Total RNA was isolated by TRI-reagent and the first strand cDNA was synthesised (Invitrogen, USA). Relative expression of *v-ATPaseA* in whitefly was measured by performing RT-PCR on ABI Prism 7300 Sequence Detection System (Applied Biosystems, USA). Three technical replicates were taken per sample. 10 µl reaction mixture contained 0.5 µl cDNA, 5 pmole primers (Table 1) and 5 µl 2× SYBR Green PCR Master Mix. Quantification was carried out by ΔΔCt quantitative method. The CT values were normalized with the corresponding values of the *actin* gene. Fold change was calculated by comparing the normalized transcript level of *v-ATPaseA* in whiteflies fed on the *v-ATPaseA* transgenic plants to that on the control (*asal*) plants.

### Statistical analyses

Statistical analyses of the leaf disc bioassay were conducted by One way-ANOVA and the means were compared using Duncan's Multiple Range Test (DMRT) with the SPSS software. The number of surviving insects in the choice assay and percent mortality in small RNA feeding bioassay were analysed for significance by Student's t-test. The difference in sugar concentration before and after whitefly infestation was also analysed for significance by Student's t-test. The t-test in all cases is against the control plant (expressing dsRNA of *asal* gene). The relative gene expression data were analyzed using 2^−ΔΔCT^ quantitation methods. The results were analyzed for significant difference using Student's t-test.

## Results

### Development of gene construct for the expression of dsRNA in transgenic tobacco targeting *v-ATPaseA* of whitefly

The gene construct in pNT4 was designed for the expression of *v-ATPaseA* specific dsRNA in transgenic plants and silence its expression in whitefly. The amplified product (189 bp) corresponds to the middle of 1842 bp long *v-ATPaseA* encoding ORF of *Drosophila melanogaster* (Figure S1). The construct was designed to express the mRNA as hairpin loop dsRNA in plants. The expression cassette was cloned in pBI101 (Figure 1A) and introduced in *A. tumefaciens* LBA 4404 for genetic transformation of tobacco.

The *asal* gene cloned from *Allium sativum* (garlic) encodes for a mannose specific lectin (A*llium sativum* leaf agglutinin). It is a storage protein and expresses in young leaves. Since ASAL is neither present in tobacco nor in whitefly, transgenic plants expressing dsRNA specific to *asal* served as negative control. It ensured that the RNAi effect was specific and accumulation of any unrelated dsRNA would not cause mortality to whitefly.

### Development of transgenic tobacco lines

Independent transgenic lines were generated which expressed hairpin RNA complementary to mRNA of *v-ATPaseA* of whitefly (eighteen lines) or the *asal* of garlic (eight lines). Integration of the gene in T_0_ transgenic lines was established by PCR. The leaf disc insect bioassay identified 12 transgenic lines of *v-ATPaseA* as causing mortality to whiteflies. These were selected for detailed study. The T_0_ lines were self pollinated and the seeds collected. The T_1_ seeds were screened for kanamycin resistance (Figure 1C). All kanamycin resistant lines showed the amplification of *v-ATPaseA+ RTM1 intron*. Eleven out of the 12 lines were positive for *nptII* gene (Figure 1D). Insect resistance in T_0_ generation, the presence of kanamycin resistance and amplification of transgene in the T_1_ generation plants established the stable integration of *v-ATPaseA*, and expression of corresponding dsRNA. The transgenic and control plants had comparable growth and morphology (Figure 1B), suggesting no specific penalty on plant development.

### Expression of *v-ATPaseA* specific dsRNA in transgenic tobacco and its processing into siRNA

**Figure 2:**
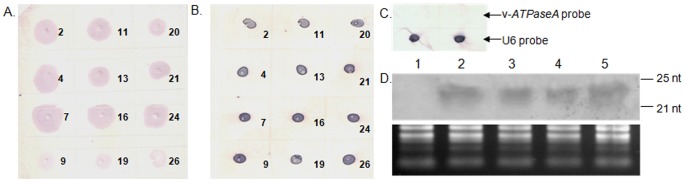
Expression analysis of v-*ATPase A* specific RNA in different transgenic lines of tobacco. Dot-blot assay of total RNA from 12 T_1_ transgenic lines with probe of (**A**) *v-ATPaseA* gene and (**B**) U6 gene, number shown are different transgenic lines. (**C**) Dot-blot assay of RNA from control plants (expressing dsRNA of *asal* gene); RNA was spotted and hybridised with same probes to show specificity of hybridisation. (**D**) Northern blot analysis of four selected transgenic lines with *v-ATP*ase*A* specific probe. Blot showed positive signal from all tested transgenic lines (Lane 2; line 7, Lane 3; line 11, Lane 4; line 16, Lane 5; line 24) while control line (Lane 1; control) did not show any signal. Loading of equal amounts of RNA was confirmed by ethidium bromide staining.

The 12 selected transgenic tobacco lines were analysed for the expression of the desired dsRNA in T_1_ generation. The dot-blot assay revealed that 9 of the 12 selected transgenic lines showed relatively higher expression as evident from the intensity of the dots (Figure 2A). The RNA from the control plants (expressing ds*asal*) showed no hybridization, indicating that the results of the dot-blot were specific to the *v-ATPaseA* gene (Figure 2C). Four lines were selected on the basis of the dot-blot assay, and analysed further by northern blot to resolve the target specific siRNA. Northern blotting confirmed the presence of siRNA corresponding to the *v-ATPaseA* in lines 4, 7, 16 and 24. The siRNA was equally abundant in all the four transgenic lines (Figure 2D). The RNA blot analysis revealed that the dsRNA was transcribed in the transgenic tobacco plants and processed by the endogenous dicer machinery into siRNA.

### Analysis of transgenic tobacco lines for whitefly resistance

Transgenic plants were evaluated for resistance against whitefly by three methods: (i) leaf disc assay, (ii) *in-vivo* bioassay of transgenic plants and (iii) bioassay with total small RNA extracted from the transgenic plants. In the leaf disc bioassay, twelve T_1_ transgenic lines were evaluated (PCR positive in T_0_ generation). After two days, 15–38% mortality was observed in the insects fed on different transgenic plants. On day four, 26–58% and on day six, 34–83% mortality was recorded (Table 2). The results showed that the transgenic line 7 gave the highest mortality of whitefly.

**Table 2:** The leaf-disc bioassay with whiteflies on transgenic tobacco lines.

Plant	Day 2	Day 4	Day 6
Control	16.55±1.00^a^	27.67±2.79^de^	29.79±1.82^e^
line 2	23.39±2.20^cd^	42.04±2.43^gh^	63.92±0.51^k^
line 4	36.37±2.09^f^	55.10±2.71^j^	81.21±0.92^m^
line 7	38.06±0.97^fg^	57.42±3.74^j^	83.58±1.36^m^
line 9	17.21±1.03^a^	27.75±2.59^de^	37.14±1.25^f^
line 11	22.76±2.59^bc^	29.04±2.61^e^	57.30±2.92^j^
line 13	23.62±2.21^cd^	38.07±1.96^fg^	58.81±2.46^j^
line 16	34.18±2.25^f^	58.72±1.67^j^	81.15±3.16^m^
line 19	15.67±1.93^a^	27.66±1.68^de^	44.55±2.15^h^
line 20	25.34±3.04^cde^	35.27±3.59^f^	64.7±1.97^k^
line 21	19.16±1.84^ab^	35.01±3.85^f^	58.55±3.72^j^
line 24	29.71±0.92^e^	49.76±5.00^i^	72.78±2.63^l^
line 26	17.03±0.57^a^	26.79±2.67^cde^	33.97±0.99^f^

Percent mortality was calculated as Average ± SD and analysed by one way ANOVA (P<0.05, df between the groups = 11, df within the groups = 24, F = 129.41). Means were compared using the DMRT at (P<0.05) (SPSS software); means superscripted with same letter within a column are not significantly different.

**Figure 3:**
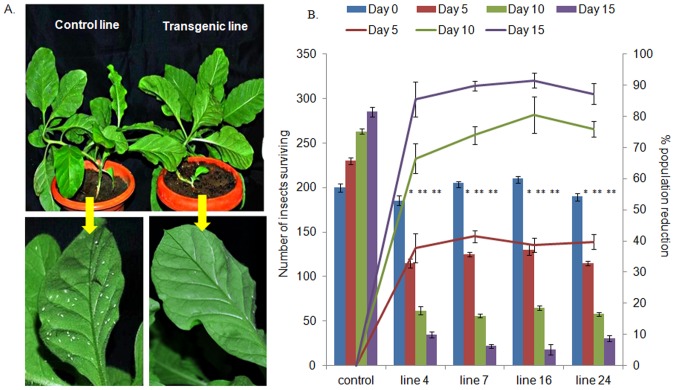
*In-vivo* bioassay of transgenic and control plants with whitefly. Control and transgenic lines challenged with freshly emerged adult whiteflies. (**A**) Whiteflies colonizing on control plant while the transgenic lines show protection, lower panel shows enlarged view of upper panel (**B**) The surviving number of insects were counted after 5, 10 and 15 days. The count of whitefly and percent population reduction was plotted for each of the selected lines. Bars = number of insects; lines = population reduction over control. Data shown are average of six plants of each line (3experimental setups ×2 plants/setup) ± standard deviation. Asterisk indicates significant difference in treatments (transgenic plants) compared to control (dsasal) plants (Student's t-test, *p<0.05,**p<0.01).

**Figure 4:**
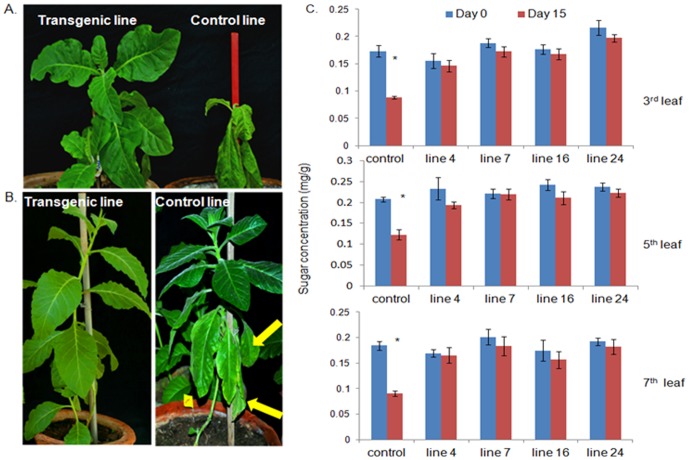
Drooping of control tobacco plants after whitefly infestation. The plants were challenged with the insects at age of 3 months (**A**) and 4.5 months (**B**). The control plants lost large amount of sap owing to heavy infestation of whitefly and drooped after 15 days, transgenic plant exhibited nearly complete tolerance. Leaves of mature plants started to droop at the base (B, indicated by arrows). (**C**) The sugar content of the leaves was estimated in the 3-month-old plants. Infestation reduced the sugar content to approximately 50% in the control plants. Asterisk indicates significant difference in sugar content after 15 days of infestation in control lines (Student's t-test, *p<0.05).

On the basis of leaf disc assay and RNA blot analysis, the *in-vivo* bioassay of transgenic plants was performed on lines 4, 7, 16 and 24. They exhibited significant protection against whitefly as compared to the control (Figure 3A). After 6 days of insect release, there was heavy infestation of whitefly on the control plants. Such plants showed the loss of vigour and drooped off. The total sugar content in leaves reduced significantly in the control plants (by ∼50%, Student's t test, p<0.05), 15 days after feeding. In the case of transgenic lines, the sugar content was not altered significantly and the infestation of whiteflies was very less (Figure 4C). In another setup, the insects were released on 4.5- month-old transgenic and control plants. The drooping effect was more pronounced in the younger plants (3-month-old). Entire plant drooped off in the previous setup, but in the older plants, only the lower leaves drooped (Figure 4A, 4B). The population of whiteflies decreased on the transgenic plants whereas their number increased rapidly on the control plants. Only 10–15% of the initial population survived on the transgenic lines (Figure 3B). Maximum population reduction was recorded in the line 16.

**Figure 5:**
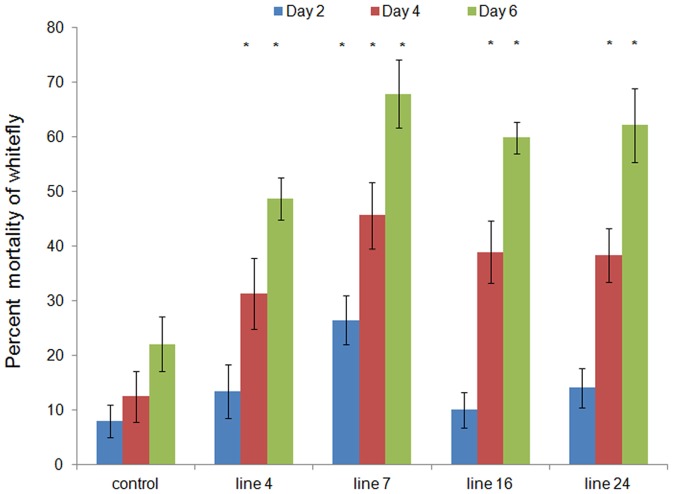
Insect bioassay with small RNAs. Small RNAs (15 µg) was fed to whiteflies after mixing in artificial diet. Graph shows percent mortality of each line (3 biological replicates ×3 technical replicates each) means ± standard deviations. Asterisk indicates significant difference in treatments (transgenic small RNA fractions) compared to control (control small RNA fractions) (Student's t-test, *p<0.05).

In order to establish the role of recombinant dsRNA in the insecticidal activity, the bioassay was performed with the small RNA preparations from transgenic and control plants. The small RNA prepared from the transgenic lines caused 48–62% mortality which was significantly higher as compared to that by the control (Figure 5, Student's t test, p<0.05). Line 7 showed the highest mortality.

### Transcript level of *v-ATPaseA* is reduced in whitefly after feeding on transgenic tobacco

**Figure 6:**
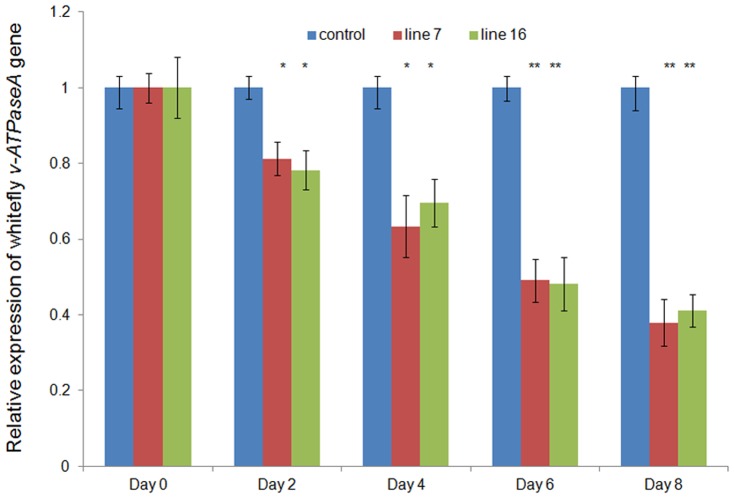
Silencing of whitefly *v-ATPaseA* after feeding on transgenic tobacco by qRT-PCR. Graph shows significant down-regulation of *v-ATPaseA* gene of whiteflies after feeding on transgenic lines. Data shown are means ± standard deviations of each line. Asterisk indicates significant difference in treatments compared to control (Student's t-test, *p<0.05, **p<0.01,).

The transcript levels of the target gene decreased significantly in whiteflies fed on the transgenic plants in comparison to those fed on the control plants (Figure 6). Lines 7 and 16 were evaluated for analysis of transcript knockdown as they showed the presence of *v-ATPaseA* specific siRNA (northern blot analysis) and also performed better in whitefly toxicity assays. The transcripts were knocked down significantly within 2 days of feeding (Student's t test, p<0.05). It dropped by 62% of the original in 8 days which was highly significant (Student's t test, p<0.01).This confirmed that the *v-ATPaseA* targeting dsRNA expressed in transgenic plants, interrupted transcription of the target gene in the insect and caused mortality. The results demonstrate that after feeding on the dsRNA expressing tobacco plants, RNAi pathway was triggered, resulting in knockdown of the target transcripts.

## Discussion

RNAi approach is increasingly being reported as useful for the control of insect pests in a variety of host plants [21]. Various research groups have provided proof-of-concept to manage crop insects through RNAi mediated silencing of the vital genes. However, this is the first report of RNAi mediated gene silencing in whitefly through feeding on transgenic plants.

Transgenic plant-mediated delivery of dsRNA in the insect gut may cause a wide range of disturbances in insect physiology, ranging from moulting defects to death [23], [24], [25]. The applicability of RNAi has not been sufficiently explored for the control of sap-sucking pests. The control of the third instar nymphs of the *Nilaparvata lugens* (brown plant hopper) was reported by interfering with the expression of three highly expressed genes of midgut through feeding on the transgenic rice expressing the respective siRNA [26].The control of *M. persicae* (green peach aphid) has also been reported through RNAi against the genes expressed in gut and salivary glands. In this case, the expression of the target transcripts was reduced by approximately 60% [27]. The silencing of genes via RNAi has been demonstrated to occur in whitefly following the injection of long dsRNA into its body cavity [28]. However, physical or chemical agents that may deliver dsRNA in whitefly under field conditions are not known. Hence, the delivery of siRNA through feeding on transgenic plants can be a relatively straight forward and effective approach to control insect pests [24], [25], [26], [27]. In the present investigation, we established *v-ATPaseA* as an important target for controlling whitefly. Middle portion of the gene, representing about 10% of the ORF, gave effective control.

The dsRNA was expressed in transgenic tobacco under the control of CaMVE35S promoter for two reasons: (a) it expresses constitutively till flowering and (b) it gives expression in companion cells. This can ensure continuous synthesis of dsRNA and its loading in sieve elements [35], [36].It is notable that the RNA moves across the plant through phloem after getting transported by the sieve elements [37].There were integration dependent variations in dsRNA expression in the independently developed transgenic lines. Though the expression of dsRNA did not affect the morphology and the life cycle of the transgenic lines, it would be important and advisable to express dsRNA temporally and also tissue specifically through phloem specific-insect bite inducible promoters [38]. This can avoid unnecessary synthesis of bio-molecules and their release in the environment and thus save plant resources.

In the *in vivo* insect challenge experiments, whiteflies preferred to feed on the control plants rather than the transgenic lines. The siRNA expressed in the transgenic plants caused some adverse physiological responses in the whiteflies, leading to their avoidance in feeding on such plants. It will be interesting to examine in future if the insects moved away from the transgenic plants with their own experience or through some signalling.

The insecticidal activity in low molecular weight RNA preparation from transgenic plants established the manifestation of RNAi. Apparently, the hairpin dsRNA was diced by the endogenous dicer machinery of the plants, resulting in formation of the 21–23 bp siRNA which knocked down the expression of target gene and thus caused mortality. Since subunit A is crucial for the activity of v-ATPase enzyme, silencing its expression killed whiteflies in a short span of time Heavy infestation of whiteflies caused severe losses of sap fluids in the control plants. Infestation in younger age caused complete drooping in the plants. As the mature plants are comparatively robust, drooping effect in them was less prominent and observed in lower leaves only. Sugar concentration of the leaves was reduced significantly. Whiteflies might cause other vital losses to host plants, like minerals, amino acids, secondary metabolites, etc. which were not evaluated in this study. Ingestion of dsRNA/siRNA might have affected the fecundity, development time and other physiological processes in whiteflies which would be evaluated in future.

Our findings are important because no GM technology for effective control of whiteflies is currently available. RNAi based approach can complement Bt-technology and thus, provide resistance against a broad variety of insect pests. The RNAi approach can be used against all the pests in principle, and allows a plethora of target genes to be silenced for effective mortality. Transgenic crops expressing functional RNAs which do not make protein are expected to have wider acceptance in comparison to the transgenes that make proteins. Products such as the Flavr-Savr tomato are examples of transgenic crops that were deregulated for commercial cultivation, and expressed nucleic acids (anti-sense technology) rather than alien proteins [39].

Our study provides proof-of-concept that plants expressing dsRNA, targeting crucial genes of whitefly, can resist its attack. Successful application of this technology would depend on several parameters such as the selection of the target gene, most effective site within the gene for knockdown, length of the targeted nucleotide sequence, and minimisation of the off-target effects. Careful consideration of the interplay of these factors is expected to deliver a competent technology in the near future.

## Supporting Information

Figure S1 **Sequence of** ***v-ATPaseA*** **gene fragment of whitefly.** (A) The sequence of *v-ATPaseA* gene was amplified from whitefly cDNA (189 bp) and used for the development of dsRNA expression cassette in transgenic tobacco lines. (B) Alignment of whitefly *v-ATPaseA* with *Drosophila melanogaster* (gi|19527546). The amplified fragment shows homology to middle region of ORF of *D. melanogaster*.
(DOCX)

Figure S2 **Leaf disc bioassay of whitefly.** (A) and (B) caps of bioassay filled with 1% agar.(C) whiteflies collected in bioassay vial.(D) Replacing the caps of vials with leaf disc containing vials.(E) Whitefly feeding on tobacco leaves.(F) Transgenic leaf disc.(G) Control leaf disc.
(DOCX)
