# Supplementary material for: Enhanced Whitefly Resistance in Transgenic Tobacco Plants Expressing Double Stranded RNA of v-ATPase A Gene
Source: PLoS One. 2014 Mar 3;9(3):e87235. doi: 10.1371/journal.pone.0087235 (PMC3940430; doi:10.1371/journal.pone.0087235)
Supplement: Figure S1 — Sequence of v-ATPaseA gene fragment of whitefly. (A) The sequence of v-ATPaseA gene was amplified from whitefly cDNA (189 bp) and used for the development of dsRNA expression cassette in transgenic tobacco lines. (B) Alignment of whitefly v-ATPaseA with Drosophila melanogaster (gi|19527546). The amplified fragment shows homology to middle region of ORF of D. melanogaster. (DOCX) [file pone.0087235.s001.docx]

(A)

CTGAAGCCTGAGAGAAATTTCCGGACGTTTGGCAGAGATGCCTGCGGACAGCGGTTACCCTGCATATTTAGGAGCTAGATTAGCTTCCTTCTACGAAAGAGCCGGTCGAATTAAGTGTCTCGGAAATCCCGACAGAGAAGGTTCTGTGAGTATTGTTGGCGCGGTATCGCCTCCTGGTGGTGACTTCTC

(B)

gi|19527546|gb|AY084150.1| GATATGGGTTACAACGTGTCCATGATGGCTGATTCCACCTCCCGTTGGGC 1200

V-ATPaseAsubunit ----------------------------CTGA--------------AGCC 8

**** * *

gi|19527546|gb|AY084150.1| TGAGGCTCTTCGTGAAATTTCTGGTCGTCTCGCTGAGATGCCTGCCGATT 1250

V-ATPaseAsubunit TGAGA--------GAAATTTCCGGACGTTTGGCAGAGATGCCTGCGGACA 50

**** ******** ** *** * ** *********** **

gi|19527546|gb|AY084150.1| CCGGCTACCCAGCCTACTTGGGAGCCCGTCTGGCCTCCTTCTACGAGCGT 1300

V-ATPaseAsubunit GCGGTTACCCTGCATATTTAGGAGCTAGATTAGCTTCCTTCTACGAAAGA 100

*** ***** ** ** ** ***** * * ** *********** *

gi|19527546|gb|AY084150.1| GCCGGTCGCGTTAAGTGCTTGGGTAACCCCGAGCGCGAGGGATCCGTGTC 1350

V-ATPaseAsubunit GCCGGTCGAATTAAGTGTCTCGGAAATCCCGACAGAGAAGGTTCTGTGAG 150

******** ******* * ** ** ***** * ** ** ** ***

gi|19527546|gb|AY084150.1| CATTGTCGGAGCTGTGTCTCCTCCTGGTGGTGACTTCTCCGATCCCGTGA 1400

V-ATPaseAsubunit TATTGTTGGCGCGGTATCGCCTCCTGGTGGTGACTTCTC----------- 189

***** ** ** ** ** ********************
