# Supplementary material for: Enhanced Whitefly Resistance in Transgenic Tobacco Plants Expressing Double Stranded RNA of v-ATPase A Gene
Source: PLoS One. 2014 Mar 3;9(3):e87235. doi: 10.1371/journal.pone.0087235 (PMC3940430; doi:10.1371/journal.pone.0087235)
Supplement: Supplementary file 2 [file pone.0087235.s002.docx]

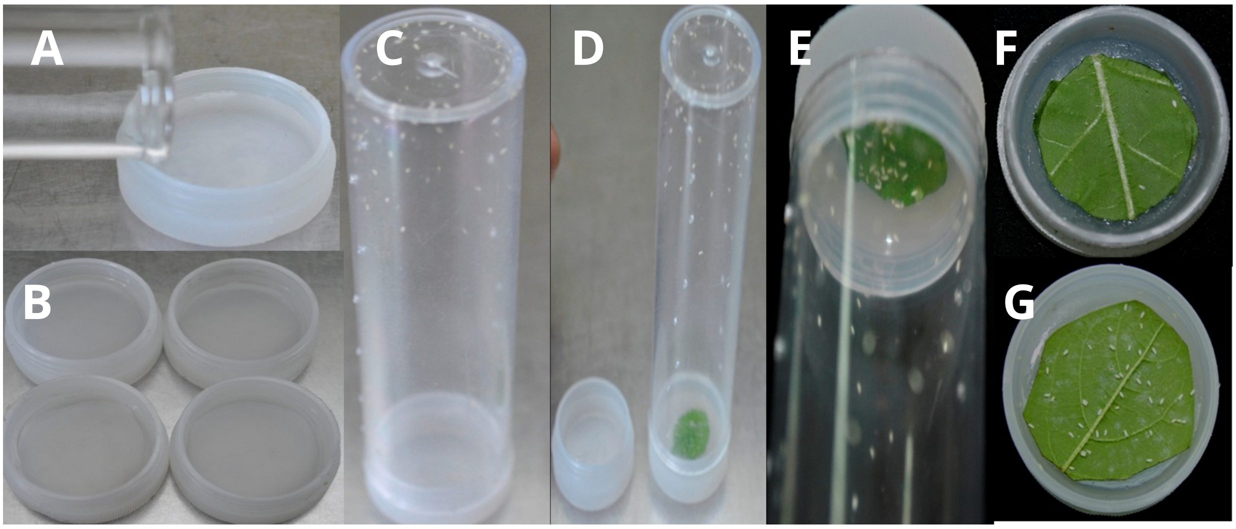
A
B
C
D
E
F
G
